# RE: Proteomic Exploration of Potential Blood Biomarkers in Haemophilic Arthropathy

**DOI:** 10.1002/hsr2.70226

**Published:** 2024-11-29

**Authors:** Quang D. La, David F. Lo, Don D. Shamilov

**Affiliations:** ^1^ Department of Biology Blinn College Bryan Texas USA; ^2^ Department of Medicine Rowan University School of Osteopathic Medicine Stratford New Jersey USA; ^3^ School of Arts and Sciences Rutgers University New Brunswick New Jersey USA

We wish to convey our reflections on the scholarly article titled “Proteomic exploration of potential blood biomarkers in haemophilic arthropathy” [1]. This investigation elucidates significant results concerning the plasma proteome of individuals afflicted with hemophilic arthropathy (HA) across a spectrum of severities, accentuating pivotal variations in protein expression correlated with disease severity and emphasizing the intricate nature of HA pathophysiology.

The researchers have discerned a range of differentially expressed proteins (DEPs) associated with inflammation and immune modulation, with particular emphasis on the heightened expression of cathepsin G (CTSG) in instances of severe HA. This observation is consistent with prior research that correlates CTSG with cartilage degradation and inflammatory responses in analogous arthritic disorders, indicating its prospective involvement in the advancement of HA. The publication titled “Cathepsin G and Its Role in Inflammation and Autoimmune Diseases” highlights the role of CTSG in inflammatory signaling pathways and its impact on cartilage deterioration in ailments such as rheumatoid arthritis (RA) [2]. It accentuates the importance of CTSG as a promising biomarker and a potential therapeutic target, positing that analogous mechanisms might be operative in HA. This correlation may facilitate the development of innovative treatment modalities aimed at modulating inflammatory responses and safeguarding cartilage integrity.

Furthermore, the research elucidates the divergent functions of a variety of proteins, encompassing the pro‐inflammatory S100‐A9 and the protective insulin‐like growth factor 1 (IGF‐1). The results pertaining to the upregulation of apolipoprotein(a) (Apo(a)), which seemingly inhibits pro‐inflammatory cytokines, are particularly significant as they correspond with the intricate immune responses delineated in the proteomic analysis of HA presented in the original article. These observations may suggest the existence of a systemic defense mechanism that seeks to mitigate disease progression.

The participation of S100‐A9 in a plethora of inflammatory disorders is of considerable importance, as it serves a crucial function in altering the phenotypic characteristics of immune cells, including neutrophils, macrophages, and dendritic cells. Recent research suggests that neutrophils lacking S100A9 demonstrate diminished cytokine secretion following stimulation via Toll‐like receptors (TLR), whereas dendritic cells deficient in S100A9 display an intensified release of cytokines [3]. This divergent response elucidates S100‐A9's potential dual role as both a pro‐inflammatory agent and a regulatory component, accentuating its significance in HA and other inflammatory pathologies. A deeper exploration into these functionalities may position S100‐A9 as a promising therapeutic target in the management of HA.

The discourse surrounding the downregulation of osteopontin (OPN) and pregnancy zone protein (PZP) in patients with hyperuricemia arthritis (HA) is particularly noteworthy. Although heightened concentrations of these proteins have been correlated with various inflammatory arthritides, their diminished expression in HA prompts inquiries regarding the distinct pathophysiological mechanisms that are operational in this specific condition. The divergent functions of these proteins in comparison to S100‐A9 may illuminate the intricate immune landscape characterizing HA and warrant further investigation into their particular roles in the disease's pathology.

An examination of the differential levels and functional roles of OPN and PZP in the context of HA vis‐à‐vis other arthritic disorders may yield significant insights into the distinct pathophysiological processes involved. OPN, a protein derived from bone tissue, has been implicated in the facilitation of joint and cartilage degradation in conditions such as RA and osteoarthritis (OA). Its diverse contributions to the immune response, along with its participation in bone remodeling, highlight its potential significance in the pathology of HA. The results indicate that, although OPN generally enhances inflammatory mechanisms in various arthritides, its diminished expression in HA could suggest alternative underlying mechanisms that influence the severity of the disease [4].

Nevertheless, it is imperative to take into account the limitations recognized by the authors of the initial investigation, including the single‐center methodology and considerable age disparities among the HA patient cohorts, which may impact the proteomic profiles recorded. These variables could potentially obscure the findings, underscoring that age‐dependent fluctuations in protein expression might affect the discerned differences in disease severity. Moreover, the emphasis of the study on plasma proteomics may neglect crucial localized joint alterations; an exploration of synovial fluid could yield a more holistic comprehension of the inflammatory milieu in HA. This methodological perspective may elucidate how proteins such as OPN and PZP contribute to the intricate nature of the disease and guide forthcoming therapeutic approaches. Considering that the original manuscript examines proteomic alterations in plasma, it would be advantageous to juxtapose these results with localized joint modifications as observed in synovial fluid, thereby enriching our understanding of HA pathophysiology.

The importance of synovial fluid in joint health cannot be overstated, as it serves a critical role in reducing friction between articular cartilages during movement. Recent literature highlights the significance of hyaluronan, a high‐molar‐mass glycosaminoglycan, in maintaining the viscosity of synovial fluid, which is essential for its lubricating properties. However, factors such as inflammation and oxidative stress can lead to the degradation of hyaluronan, potentially exacerbating joint diseases, including HA [5]. This suggests that analyzing synovial fluid proteomics could provide valuable insights into local joint pathophysiology and help identify biomarkers indicative of disease progression.

In conclusion, the findings from the research on HA not only highlight the complex interplay of molecular factors involved in its pathophysiology but also open avenues for potential biomarkers that may aid in disease management. The contrasting expressions of proteins like S100‐A9, osteopontin, and pregnancy zone protein suggest a unique immune landscape in HA that differs from other arthritic conditions. Further studies are essential to validate these initial findings and to investigate the specific contributions of synovial fluid proteomics, which may enhance our understanding of the inflammatory processes at play and inform future therapeutic strategies.

## Author Contributions

**Quang D. La:** writing–original draft, conceptualization. **David F. Lo:** writing–review and editing, supervision, validation. **Don D. Shamilov:** validation; writing–review and editing, supervision.

## Conflicts of Interest

The authors declare no conflicts of interest.

## Transparency Statement

We, the authors of this study, affirm that this manuscript is an honest, accurate, and transparent account of the study being reported, that no important aspects of the study have been omitted, and that any discrepancies from the study as planned (and, if relevant, registered) have been explained.

## Data Availability

The data that support the findings of this study are available from the corresponding author upon reasonable request.
